# Moxibustion for gastrointestinal cancer-related fatigue: study protocol for a randomized clinical trial

**DOI:** 10.3389/fpsyt.2026.1751757

**Published:** 2026-05-11

**Authors:** Huanyi Liu, Qian Fan, Wang Yao, Lumin Liu, Junwei Hu, Ping Yin, Yuelai Chen

**Affiliations:** Sleep Medicine Centre, LongHua Hospital Affiliated to Shanghai University of Traditional Chinese Medicine, Shanghai, China

**Keywords:** cancer-related fatigue, complementary and alternative medicine, gastrointestinal cancer, moxibustion, placebo moxibustion

## Abstract

**Background:**

Cancer-Related Fatigue (CRF) is one of the most prominent subjective symptoms that cancer patients encounter, in particular individuals with gastrointestinal tumors. This fatigue significantly impacts their quality of life, impairs adherence to treatment, and may ultimately reduce overall survival rates. Moxibustion has gradually gained recognition as a therapeutic intervention for CRF. However, clinical evidence supporting the efficacy of moxibustion remains limited.

**Methods:**

This investigation employs a randomized, controlled, single-blinded trial design. A 1:1 random allocation method will be implemented to assign 74 participants to either the moxibustion group or the placebo moxibustion group. All participants will undergo eight sessions over 4 weeks, with sessions occurring twice weekly and lasting for 30 minutes each. Assessments for follow-up will take place at week 8. The primary outcome will be the percentage of patients who show a decrease of at least three points on the Brief Fatigue Inventory (BFI) at week 4. Secondary outcomes will encompass changes in the BFI, Quality of Life Questionnaire-Core 30 (QLQ-C30), Generalized Anxiety Disorder Scale-7 (GAD-7). Meanwhile, Blood cell counts and serum T lymphocyte subsets (CD3+, CD4+, CD8+, CD4+/CD8+) will be measured as exploratory outcomes. In addition, adverse events will be screened and documented throughout the study. An analysis adhering to the intention-to-treat(ITT) principle will be executed.

**Discussion:**

This research aims to evaluate the efficacy and safety of moxibustion in patients with gastrointestinal CRF. Additionally, the study seeks to investigate the effects of moxibustion on the immune response in individuals diagnosed with gastrointestinal CRF.

## 1 Introduction

The International Agency for Research on Cancer (IARC) reported in the ‘Global Cancer Statistics 2020’ that colorectal and gastric cancers are the third and fifth most frequently diagnosed malignancies globally (1). The advancement of diagnostic and therapeutic technologies has led to an increase in overall survival rates for individuals with gastrointestinal cancers. However, a troubling reality remains: studies suggest that a substantial proportion of patients between 52% to 90% either undergoing treatment or existing as survivors, endure an unyielding level of fatigue that resists conventional rest, a condition known as CRF (2). CRF has emerged as a pervasive and deeply felt symptom among cancer patients. A sweeping meta-analysis, amalgamating data from 129 studies and encompassing 71,568 cancer patients, has unveiled disparities in fatigue prevalence across various cancer subtypes. Most notably, gastrointestinal cancer patients report the most severe fatigue levels, hitting a high of 50%, which is considerably higher than those observed in gynecological (26.2%) and prostate (26.3%) cancer patients (3).

The 2023 second edition of the National Comprehensive Cancer Network (NCCN) guidelines defines CRF as a persistent, debilitating, and subjective experience of physical, emotional, and cognitive fatigue linked to the presence of cancer or its therapeutic interventions. This type of fatigue is disproportionate to recent activity levels and disrupts daily functioning (4). It is a more profound and enduring condition than the fatigue that follows physical exertion and is not alleviated through rest or sleep. CRF can have a profound impact on the physiological, psychological, and socioeconomic well-being of both patients and their primary caregivers, potentially diminishing quality of life, hindering treatment adherence, and impacting overall patient survival (5–7). Consequently, alongside treating the primary gastrointestinal cancer, attention must also be given to the management of fatigue symptoms and the enhancement of life quality for these patients.

Currently, there is a lack of widely accepted and efficacious pharmaceutical treatments for CRF. The NCCN endorses a range of non-pharmacological strategies, including exercise-based therapies, cognitive-behavioral interventions, sleep hygiene practices, and psychological support, which have shown potential in alleviating CRF (8), although their benefits may vary. With a significant number of patients experiencing CRF, there is a growing interest in complementary and alternative medicine (CAM) options (9). Moxibustion, a traditional CAM therapy involving the application of heat from burning herbs like mugwort to specific acupoints on the body. This treatment modality operates through direct or indirect thermal stimulation across a range of temperatures (10, 11). Moxibustion, appreciated for its straightforward application, non-invasive approach, and significant healing properties, has been employed in China for the management of CRF. Nevertheless, there is a lack of high-quality clinical trials to support the therapeutic benefits of moxibustion for CRF. The field is calling for rigorous, methodologically sound randomized controlled trials to ascertain the efficacy of moxibustion in treating CRF.

In response to these needs, we have crafted a randomized controlled trial aimed at assessing the efficacy and safety of moxibustion in treating gastrointestinal CRF and at exploring its potential effects on the immune function of patients afflicted with this condition.

## 2 Methods

### 2.1 Study design

**Figure 1:**
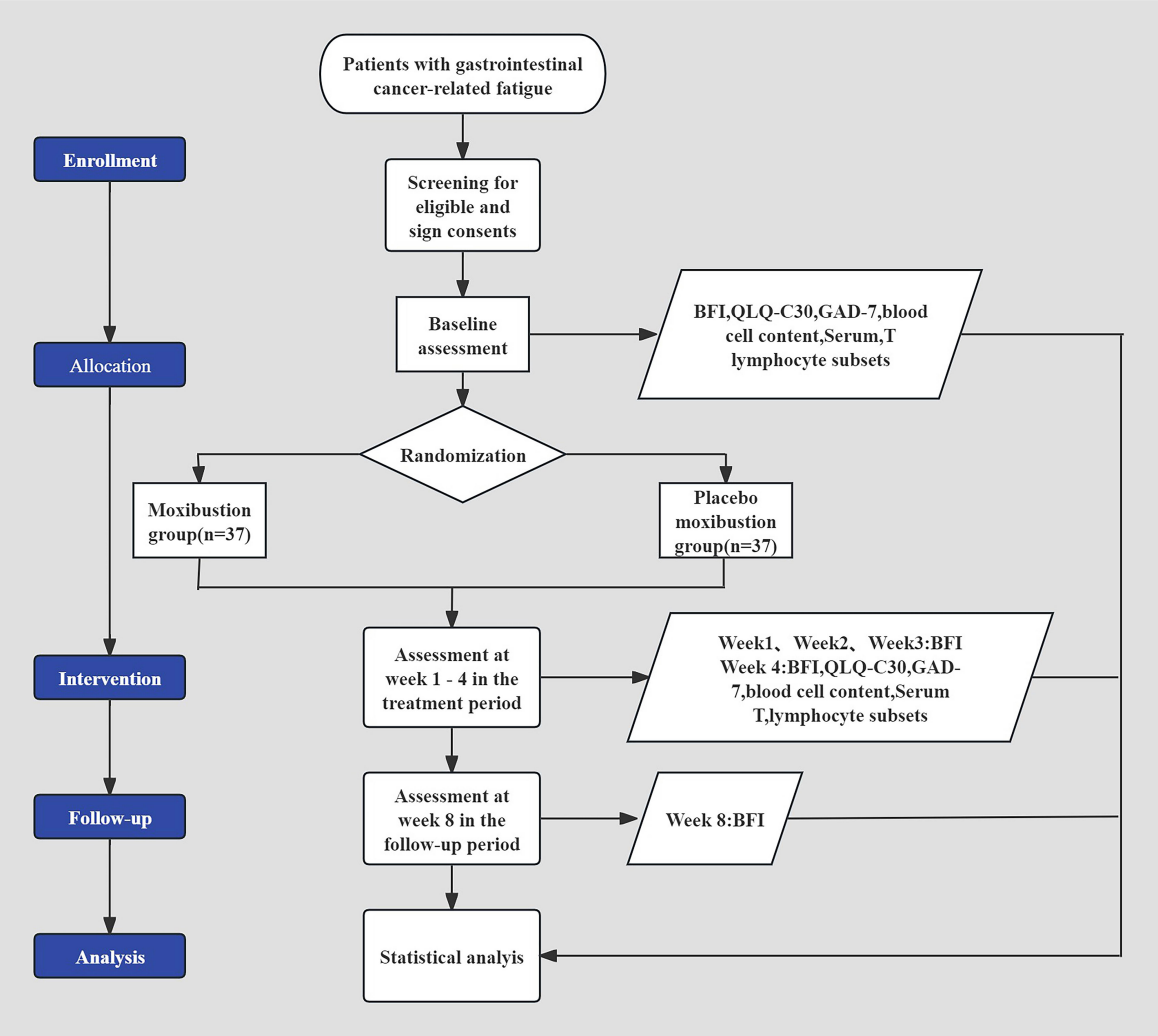
Flowchart of the study. BFI: the Brief Fatigue Inventory; QLQ-C30: Quality of Life Questionnaire-Core 30; GAD-7: Generalized Anxiety Disorder Scale-7; Blood cell counts (including leukocytes, hemoglobin, platelets, and lymphocytes);Serum T lymphocyte subsets (including CD3+, CD4+, CD8+, CD4+/CD8+).

The LongHua Hospital affiliated to Shanghai University of Traditional Chinese Medicine will host this randomized, single-blinded, placebo-moxibustion controlled study. 74 patients who satisfy the gastrointestinal CRF inclusion requirements will be divided 1:1 between the moxibustion and placebo moxibustion groups at random. The trial is structured to include a 4-week intervention segment followed by a subsequent 4-week post-intervention surveillance phase. This report was based on the Standard Protocol Items: Recommendations for Interventional Trials checklist (12). This protocol is version 1.0. **Figure 1** shows a flowchart outlining the specifics of the trial’s procedure. **Table 1** offers a timetable that includes baseline, interventions, and evaluations.

**Table 1:** Schedule of baseline, interventions, and assessments.

Study period	Baseline	Intervention phase				Follow-up phase
Time point	Week-1- 0	Week 1	Week 2	Week 3	Week 4	Week 8
Enrollment	•					
Eligibility screen	•					
Informed consent	•					
Medical history collection	•					
Randomization	•					
Intervention						
Moxibustion		•	•	•	•	
Placebo moxibustion		•	•	•	•	
Primary outcome						
percentage of patients showing a decrease of at least three points on the BFI at week 4					•	
Secondary outcomes						
BFI	•	•	•	•	•	•
QLQ-C30	•				•	
GAD-7	•				•	
Exploratory outcomes						
Blood cell counts	•				•	
serum T lymphocyte subsets	•				•	
Adverse events		•	•	•	•	

### 2.2 Patient recruitment

The cancer wards and outpatient clinics of LongHua Hospital affiliated to Shanghai University of Traditional Chinese Medicine will be the source of gastrointestinal CRF patients. Posters and announcements will be posted on official platforms at the same time. For a preliminary screening, those who are interested might call the researcher at the number listed on the website. An impartial researcher will conduct an initial evaluation and in-person interview with those who first meet the requirements. After signing an informed consent form, those who fulfill the inclusion requirements will be assigned at random to either the moxibustion or placebo moxibustion groups. Personal data will be used solely for medical research, and each participant will have the freedom to leave the trial at any time.

### 2.3 Eligibility criteria

#### 2.3.1 Inclusion criteria

- Prior to providing written informed consent for study participation, subjects must meet all of the following diagnostic criteria:
- Diagnosis of colorectal and gastric cancer according to the 2022 Chinese Clinical Society of Oncology’s (CSCO) guidelines.
- The tumor node metastasis (TNM) stage II or III based on the 8th edition of the American Joint Committee on Cancer (AJCC) Staging Manual.
- Fulfill the CRF diagnostic criteria outlined in the International Classification of Diseases (ICD-10).
- CRF occurred after the diagnosis of colorectal and gastric cancer and is linked to the cancer or its treatment.
- No gender restriction, age 18-70.
- The performance status of the Eastern Cooperative Oncology Group (ECOG) ≤ 2.
- BFI≥4.
- Prediction of survival ≤ 6 months.
- No previous Acupuncture or moxibustion treatment within the least 3 months.
- Possess normal cognitive function, capable of comprehending scale instructions and completing the assessment.
- Participation and signed informed consent are required.

#### 2.3.2 Exclusion criteria

- Presence of serious diseases affecting the heart, liver, kidneys, or other major organs.
- Patients scheduled to undergo surgery, radiotherapy, or chemotherapy during the trial period.
- Pregnant or lactating women.
- Presence of intestinal perforation, hemorrhage, obstruction, or any other contraindications to moxibustion.
- Individuals with psychiatric conditions or substance abuse (drugs, alcohol, medications) compromising communication.
- Patients with ulcers, sores, or localized skin infections at the proposed moxibustion sites.
- Patients with respiratory diseases, a known allergy to moxibustion, or any other conditions for which moxibustion therapy is contraindicated.

### 2.4 Randomization

The SAS 9.4 (SAS Institute Inc., Cary, NC) software’s “Proc Plan” process will generate random numbers that will be used to divide the patients into two groups. After being sealed in envelopes, the random allocation cards will be opened one after the other according to the patients’ visits. Based on the cards in the envelopes, the researcher will divide the eligible individuals in a 1:1 ratio between the moxibustion group and the placebo moxibustion group. Each participant will be made conscious that they have an equal probability of being placed in either group.

### 2.5 Blinding

In the course of the study, all participants, including subjects, efficacy evaluators, data analysts, and statistical analysts, with the exception of the acupuncturists, will be blinded to the group assignments. To preserve this blinding, rigorous measures will be enacted to limit the exchange of relevant information among participants. These measures include the use of separate treatment rooms and the requirement for participants to wear eye masks. At the end of the 4-week treatment period, blinding assessment will be conducted using a questionnaire. Patients will be asked to respond to the question: “Which treatment do you believe you received?” with options including “Moxibustion, Placebo moxibustion, or Uncertain.” The evaluation will be performed using Bang’s Blinding Index(BI) system for quality control of blinding, with the BI for each group calculated through a mathematical model. BI will be used to evaluate blinding quality. Values close to 0 indicate successful blinding, while values further from 0 suggest potential unblinding. The BI results for each group will be interpreted descriptively to assess the adequacy of blinding.

### 2.6 Interventions

#### 2.6.1 Moxibustion group

**Figure 2:**
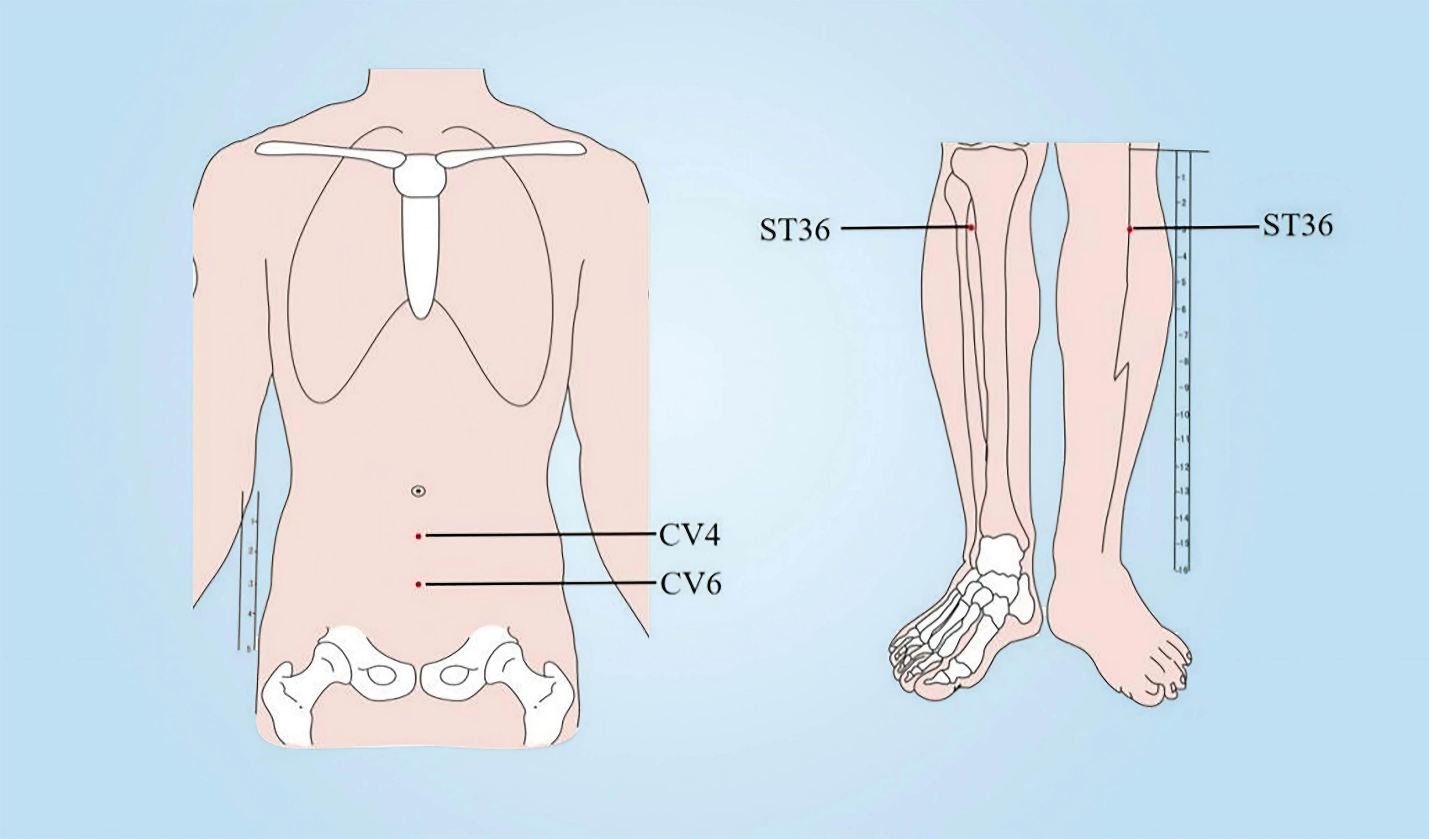
Location of acupoints in the moxibustion group and placebo moxibustion group. CV 4: Guan Yuan; CV6: Qi Hai; ST 36:Zu San Li.

**Figure 3:**
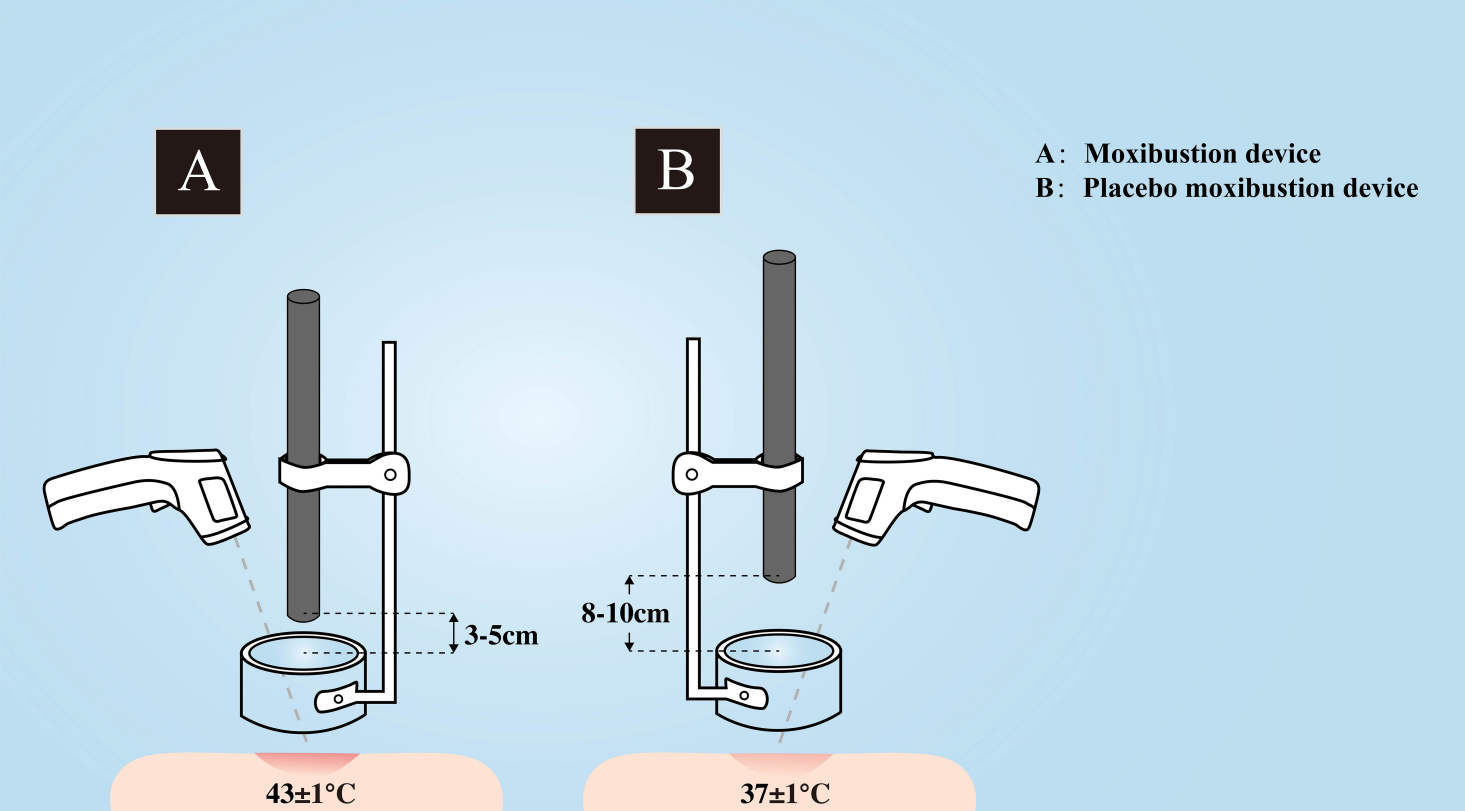
Moxibustion and placebo moxibustion device. **(A)** Moxibustion device; **(B)** Placebo moxibustion device.

Patients allocated to the intervention group will receive moxibustion therapy targeting the midline acupoints Guan Yuan (CV4) and Qi Hai (CV6), together with bilateral Zu San Li (ST36) acupoints in a supine position. The acupoint locations are determined based on the The People’s Republic of China’s State Standard (GB/T 12346-2021). Detailed illustrations of the acupoint locations are provided in **Figure 2** and **Table 2**. In the moxibustion group, patients will be treated with pure moxa sticks (diameter: 2.8 cm; Hanyi, Nanyang, China) ignited and placed on a stand to maintain 3–5 cm from the acupoints. The skin temperature at the acupoints will be regulated at 43 ± 1 °C and monitored using an infrared thermometer (Fluke 62, Fluke Corporation, Everett, WA, USA) (13). The moxibustion device used in the study is shown in **Figure 3**.Two 30-minute sessions of treatment will be offered twice a week for a total of eight sessions spread over four weeks. During treatment, the burning moxa stick will be monitored continuously to maintain a stable thermal stimulus. Ash will be removed promptly as needed to ensure consistent combustion and to avoid excessive heat fluctuation or ash dropping during the procedure.

**Table 2:** The location of acupoints in the moxibustion group and placebo moxibustion group.

Acupoint	Location
Zu San Li(ST36)	On the outer side of the lower leg, 3 *cun* below the outer knee, and one horizontal finger’s breadth away from the anterior edge of the tibia.
Guan Yuan(CV4)	On the lower abdomen midline, 3 *cun* below the umbilicus.
Qi Hai(CV6)	On the lower abdomen midline, 1.5 *cun* below the umbilicus.

*cun*: a proportional body measurement used in acupuncture; one horizontal finger’s breadth: the width of the patient’s thumb at the interphalangeal joint, or a proportional digital measurement depending on your standard source.

#### 2.6.2 Placebo moxibustion group

Patients allocated to the placebo moxibustion group will undergo treatment at identical acupoints as those in the moxibustion group, using the same type of moxa sticks. However, the placebo moxibustion will be applied at a distance of 8–10 cm from the acupoints to maintain a temperature of 37 ± 1 °C. The device utilized for the placebo moxibustion is depicted in **Figure 3**. Additionally, the duration and number of sessions for the placebo moxibustion treatment will mirror those of the moxibustion group.

#### 2.6.3 Management of concomitant anticancer treatments

To minimize potential confounding effects on fatigue severity and immune-related outcomes, patients who are scheduled to undergo surgery, radiotherapy, or chemotherapy during the intervention period will not be enrolled in this trial. At baseline, detailed information on prior and current anticancer treatments will be collected, including surgery, chemotherapy, radiotherapy, targeted therapy, immunotherapy, and supportive care, as well as the interval since the last anticancer treatment. During the study period, any clinically relevant treatment changes or medical events that may affect fatigue or immune status, such as the initiation of new anticancer therapy, treatment escalation, infection, blood transfusion, or the use of hematopoietic growth factors or corticosteroids, will be documented in detail. These factors will be considered during data interpretation, and sensitivity or adjusted analyses will be performed if necessary to evaluate the robustness of the primary outcome.

### 2.7 Outcomes

#### 2.7.1 Medical history collections

The collected patient medical history will be data on participant demographics and medical history, including gender, age, height, weight, disease duration, tumor type, tumor stage, and treatment modalities.

#### 2.7.2 Primary outcome

The primary outcome measure is the proportion of patients achieving a reduction of at least 3 points on the BFI at week 4. A responder-based endpoint was selected because it provides a clinically interpretable estimate of the proportion of patients achieving meaningful fatigue improvement. This approach is also consistent with prior symptom-management trial in CRF, in which the BFI has been used not only as a continuous measure of fatigue severity but also to classify clinically relevant improvement or normalization of fatigue status (14). In the present trial, a 3-point reduction was prespecified as an interpretable response threshold based on prior fatigue literature, the 0–10 scaling properties of the BFI, and the preliminary data used for sample size estimation, while continuous changes in BFI scores are also analyzed as secondary outcomes (15). BFI is a subjective and reliable measure assessing the level of fatigue in the past 24h and the impact of fatigue on patients. The questionnaire consists of nine items rated on a 0–10 scale, with a final score range of 0-10. Fatigue is rated as mild (1-3), moderate (4-6), or severe (7-10) (16).

#### 2.7.3 Secondary outcomes

##### 2.7.3.1 The changes in BFI scores

It will be evaluated at weeks 1,2,3,4, 8.

##### 2.7.3.2 QLQ-C30

The quality of life (QoL) of cancer survivors is assessed using a detailed 30-item questionnaire. This instrument incorporates a global QoL scale alongside multiple functional scales that evaluate physical, role, cognitive, emotional, and social functioning. Furthermore, it features symptom scales that focus on pain, fatigue, and nausea and vomiting. The questionnaire also includes specific items that measure dyspnea, constipation, insomnia, appetite loss, diarrhea, and the financial repercussions of the illness (17). It will be assessed at weeks 0 and 4.

##### 2.7.3.3 GAD-7

The brief anxiety self-assessment tool comprises 7 items, each evaluated on a scale ranging from 0 to 3, resulting in a cumulative score that can vary from 0 to 21. Scores 5–9 suggest the presence of mild anxiety, scores 10–14 indicate moderate anxiety, and scores 15–21 are indicative of severe anxiety (18). It will be assessed at weeks 0 and 4.

#### 2.7.4 Biological specimen exploratory outcomes

Given that CRF has been associated with inflammation and immune dysregulation, exploratory biological outcomes were included to investigate whether symptom changes may be accompanied by changes in host immune status. Peripheral blood cell counts and serum T lymphocyte subsets were selected as clinically accessible exploratory indicators. Exploratory analysis of biological indicators include the following items: (1)Blood cell counts, which include leukocytes, hemoglobin, platelets, and lymphocytes. It will be evaluated at weeks 0 and 4. (2)Serum T lymphocyte subsets, which include CD3+, CD4+, CD8+, CD4+/CD8+. It will be evaluated at weeks 0 and 4.

#### 2.7.5 Safety evaluation

Adverse events (AEs) linked with moxibustion are burns, allergic reactions, and blistering, along with potential worsening of pre-existing conditions due to the therapy. In the event of any AEs, it is imperative for the practitioner to promptly assess their severity and implement suitable interventions, such as removing the heat source or consulting a dermatologist for severe burns. Any AEs noticed during the study need to be documented in the “Adverse Event Form”. It is imperative that both the management protocols and the resultant outcomes of these AEs are dutifully recorded with precision. In the event that a participant experiences serious AEs during treatment, the clinical trial shall be assessed and potentially terminated at the discretion of the researchers. Treatment termination details, including final time and outcome, will be documented for statistical analysis of AEs.

### 2.8 Data collection and management

Before initiating a clinical trial, researchers will engage in comprehensive training sessions that focus on the study protocol. To improve participant compliance, researchers must rigorously implement the process of informed consent, ensuring that participants possess a comprehensive understanding of the experimental requirements and agree to cooperate. The study will provide complimentary moxibustion treatment, conduct monitoring and evaluation of relevant indicators, offer transportation subsidies, and deliver healthcare guidance. Researchers meticulously adhere to clinical trial protocols and implement standard operating procedures to maintain rigorous quality control and ensure the effective execution of quality assurance systems in clinical trials.

The project team has established a data quality control group tasked with conducting both scheduled and unscheduled monitoring visits, as well as verifying the study’s data integrity. Clinical data for each participant will be gathered using CRFs. Following the completion of each participant’s observation session, the researcher is required to submit CRFs and informed consent documentation to the data quality control team for evaluation within three working days. During the supervision process, should any protocol deviations be identified, team members are required to complete a violation record. This record must include detailed documentation of the time of discovery, the timing and sequence of the incident, the underlying reasons, and the corresponding corrective actions. The record must be signed by the researcher and reported to the Ethics Committee in accordance with relevant regulations. In statistical and summary reports, the influence of protocol violations on the final data and conclusions is systematically analyzed, evaluated, and documented.

### 2.9 Sample size calculation

Based on the preliminary research results, it was observed that 40% of patients in the moxibustion group and 10% in the placebo group exhibited a decrease of at least 3 points on the BFI at week 4. Calculations indicate that the sample size of 58 patients would ensure a study power of at least 80%, with a two-sided significance level of 0.05. To adjust for an anticipated dropout rate of 20%, 37 patients will be finally taken from for each group.

### 2.10 Data analysis and statistical methods

SPSS 25.0 will be used for statistical analysis, with an ITT methodology. Missing data will be imputed with the last available evaluation. Continuous variables will be presented as mean ± standard deviation (SD) or as median with interquartile range[M (IQR)], while categorical variables will be expressed in terms of frequencies or percentages. Statistical significance will be determined at *p* < 0.05.Baseline anticancer treatment characteristics and clinically relevant concomitant treatments during the trial will be summarized descriptively; if needed, sensitivity analyses or adjusted analyses will be conducted to assess the robustness of the primary outcome.

The proportion of patients with a minimum 3-point reduction on the BFI at week 4 will be assessed using Chi-Square or Fisher’s exact tests for categorical data. For repeated measures, such as changes in BFI scores (week 1,2,3,4,8), Generalized Estimating Equations (GEE) will be employed.

GAD-7, QLQ-C30, leukocyte counts, hemoglobin levels, platelet counts, lymphocyte counts, and CD3+, CD4+, CD8+, and CD4+/CD8+ ratios are examples of continuous variables for which unpaired t-tests or Mann-Whitney U tests will be used for between-group comparisons, as applicable.

### 2.11 Ethics and dissemination

The trial involving humans has been approved by the LongHua Hospital affiliated to Shanghai University of Traditional Chinese Medicine (Ethical approval number: 2022LCSY048). Informed consent will be obtained from all participants before participation in the study, in accordance with local law and institutional requirements. Following the conclusion of the study, we will widely distribute our results by presenting at international and national conferences or publishing in open-access scholarly journals.

## 3 Discussion

CRF is a common and distressing symptom experienced by cancer survivors undergoing treatment, affecting their quality of life and leading to decreased treatment compliance (19). The management of fatigue symptoms in cancer patients has received increased attention from medical professionals and researchers, yet no specific treatments have been established for CRF. Moxibustion, a traditional Chinese medical therapy with a long history, is often used for its non-invasive nature and is commonly employed to address issues related to debility, fatigue, and aging (20). Moxibustion has shown promise in treating CRF, with the American Society of Clinical Oncology – Integrative Oncology Society in 2024 recommending its use in managing fatigue in adult cancer survivors after treatment completion (21). Furthermore, multiple meta-analyses indicate that moxibustion could potentially reduce CRF and enhance the quality of life in cancer patients (22–24). Nevertheless, the available evidence remains limited by heterogeneity in cancer populations, intervention protocols, and study quality, highlighting the need for further well-designed randomized controlled trials. In the present study, the acupoint prescription was selected primarily with reference to previously published clinical studies on moxibustion for CRF, in which CV4, CV6, and ST36 were repeatedly used and showed good clinical feasibility (25, 26). The treatment frequency and duration adopted in this trial were likewise informed by prior CRF studies and were chosen to balance treatment intensity with adherence and feasibility in patients receiving cancer care (9). Therefore, this single-blind, placebo-controlled trial was designed to provide a more rigorous evaluation of the efficacy and safety of moxibustion for gastrointestinal CRF.

Placebos play a crucial role in the evaluation of clinical efficacy, as they help to distinguish specific from non-specific effects and reduce bias through blinding. The setting of placebo moxibustion group has always been a challenge in clinical studies. Multiple placebo moxibustions are mainly implemented by placing a heat shield at the bottom of the moxibustion device to block smoke, heat and radiation (27–29). However, the distinct characteristics of moxibustion treatment make it difficult to fully blind participants to the intervention if thermal stimulation is prevented. The current trial utilizes a placebo moxibustion device that is visually indistinguishable from the device used in the moxibustion group. Additionally, identical moxa stick are utilized to ensure consistency in the aroma produced by the burning moxa, thereby maximizing the blinding of the patients involved in the study. This placebo moxibustion method has been validated in multiple clinical trials of moxibustion, demonstrating its effectiveness and reliability (13, 30, 31).

The degree of fatigue varies with different types of cancer. It is particularly necessary to focus on research related to fatigue associated with specific types of cancer. However, current research on the efficacy of moxibustion in treating CRF often involves a mixed variety of cancers, leading to significant heterogeneity (25, 29). Moreover, existing studies on moxibustion for CRF are predominantly concentrated on breast cancer (22, 32), with few scholars focusing on gastrointestinal CRF. It is essential to concentrate on the fatigue associated with gastric and colorectal cancers to provide a more detailed and relevant exploration. Focusing on gastric and colorectal cancer may also reduce part of the clinical heterogeneity that is commonly seen in CRF studies involving mixed cancer populations, thereby improving the interpretability of both symptom outcomes and exploratory biomarker findings.

The inclusion of exploratory immune-related outcomes in this trial was based on the growing evidence that CRF is associated not only with symptom burden, but also with inflammation and immune dysregulation. Previous reviews have suggested that inflammatory processes may contribute to the development and persistence of fatigue in patients with cancer and survivors (33). In this context, peripheral blood cell counts were included as clinically accessible indicators that may reflect anemia, systemic inflammatory status, and general immune condition relevant to fatigue. In addition, T-lymphocyte subsets, including CD3+, CD4+, CD8+, and the CD4+/CD8+ ratio, were selected as exploratory biomarkers because recent meta-analytic evidence suggests that acupuncture and moxibustion may modulate these immune parameters in patients with malignant tumors (34). In the present study, these indicators are intended to provide preliminary evidence on whether fatigue improvement may be accompanied by measurable changes in host immune status, rather than to establish a definitive immunological mechanism.

The study is subject to several limitations. Primarily, the sample size is relatively small. Additionally, the duration of the follow-up period was limited. Future investigations should aim to mitigate these limitations by utilizing larger sample sizes and extending the follow-up periods. Implementing a multicenter trial design could further enhance the diversity of the patient population and bolster the external validity of the results.

## Glossary

CRF: Cancer-Related Fatigue
BFI: Brief Fatigue Inventory
GAD-7: Generalized Anxiety Disorder Scale-7
QLQ-C30: Quality of Life Questionnaire-Core 30
ITT: Intention-to-treat
IARC: International Agency for Research on Cancer
NCCN: National Comprehensive Cancer Network
CAM: Complementary and alternative medicine
CSCO: Chinese Clinical Society of Oncology’s
TNM: Tumor node metastasis
AJCC: American Joint Committee on Cancer
ICD-10: International Classification of Diseases
ECOG: Eastern Cooperative Oncology Group
BI: Bang’s Blinding Index
QoL: Quality of life
AEs: Adverse events
SD: Standard deviation
GEE: Generalized Estimating Equations.
